# Creating Coarse-Grained Systems with COBY: Toward Higher Accuracy of Complex Biological Systems

**DOI:** 10.1021/acs.jcim.5c00069

**Published:** 2025-05-12

**Authors:** Mikkel D. Andreasen, Paulo C. T. Souza, Birgit Schiøtt, Lorena Zuzic

**Affiliations:** † Department of Chemistry, 1006 Aarhus University, Langelandsgade 140, 8000 Aarhus C, Denmark; ‡ Laboratoire de Biologie et Modélisation de la Cellule, CNRS, UMR 5239, Inserm, U1293, Universite Claude Bernard Lyon 1, Ecole Normale Supérieure de Lyon, 46 allée d’Italie, 69364 Lyon, France; § Centre Blaise Pascal de Simulation et de Modélisation Numérique, Ecole Normale Supérieure de Lyon, 6 allée d’Italie, 69364 Lyon, France

## Abstract

Current trends in molecular modeling are geared toward increasingly realistic representations of the biological environments reflected in larger, more complex systems. The complexity of the system-building procedure is ideally handled by software that converts user-provided descriptors into system coordinates. This, however, is not a trivial task, as building algorithms use simplifications that result in inaccuracies in the system properties. We created COBY, a coarse-grained system builder that can create a large variety of systems in a single command call with an improved accuracy of the complex membrane and solvent building procedures. COBY also contains features for building diverse systems in a single step, and has functionalities aiding force field development. COBY is an open-source software written in Python 3, and the code, documentation, and tutorials are hosted at https://github.com/MikkelDA/COBY.

## Graphical abstract

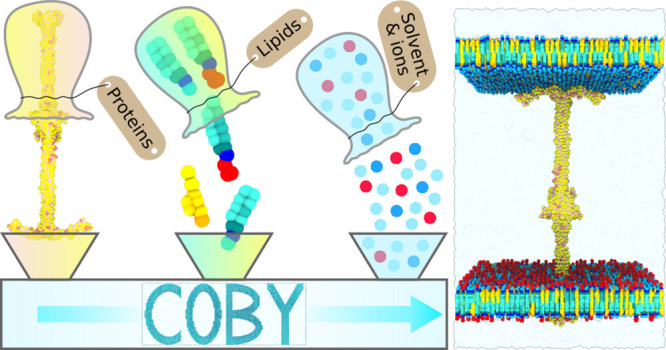

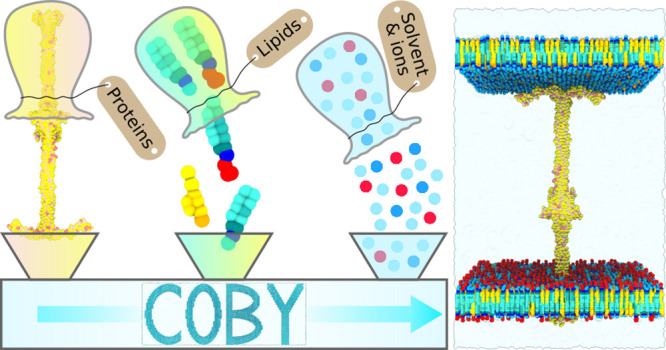

## Introduction

Dynamics and function within a biological system are inextricably linked. Molecular dynamics (MD) simulations are a computational tool used to investigate motions within a macromolecular system on microsecond time-scales and in atomistic or near atomistic resolution. By using experiment-informed structures paired with force fields defined by realistic interatomic potentials, simulations complement the experimental method and allow for a detailed view of an individual system in motion.^1^

Continuously increasing computing resources, paired with the advancements in integrative biology, makes it possible to model systems of ever greater complexity. One particular area of interest is complex membranes, as membrane composition is linked to a plethora of biological functions, such as protein regulation through lipids,^2–8^ modulated protein kinetics,^9^ protein localization,^10^ and biophysical properties of the membrane.^11^ However, computational costs of achieving adequate sampling increase dramatically with system complexity. Simulating the complex membranes in a coarse-grained (CG) resolution, where multiple atoms are grouped together to form a bead, allows for longer sampling of bigger systems and ultimately a wider exploration of the conformational landscape^12,13^ —a feat that is not easily achievable in atomistic simulations.

To run the MD simulations, one needs to start with system building, which is a user-involved process and can quickly become laborious in higher-complexity systems. The systems are therefore often built with the help of a software that automates the building process. It follows that system accuracy is directly dependent on the accuracy of the employed code.^1^

Membrane building in particular is reliant on the available software.^13^ The procedure is nontrivial, as the membrane structure is not directly sourced from a given set of experimentally determined atom coordinates (as is the case with proteins); instead, the coordinates need to be built ”from scratch”, based only on few given parameters: membrane patch size, lipid types and their ratios, and the assigned area per lipid (APL). Ideally, all of the given parameters should exact to the given value in a built membrane; however, this is often not the case. Fundamentally, a membrane built for the purpose of MD simulations can only accept a whole number of lipids, which in consequence requires nontrivial consideration of the given parameters, as rounding can lead to value drift. This problem is exacerbated in complex membranes, where each lipid ratio (both within and between the leaflets) needs to be satisfied using a finite, integer number of lipids. If not considered, the resulting ratios will be offset from the requested values and produce an inaccurate model of a complex membrane.

There exists a number of tools that can be used to automatize building of biomolecular systems (e.g., Charmm-GUI,^14–16^ TS2CG,^17^ *insane*,^18^ PACKMOL,^19^ polyply^20^), and while each offers a bespoke set of functionalities, we identified the need for an increased accuracy in terms of lipid ratios in complex membrane systems, coupled with ease of use, speed, and customization ability.

Here we introduce a new system building software tool named COBY (Complex Biological System Builder), which is an open-source Python package for building Martini coarse-grained systems.^21^ COBY was primarily designed to build flat complex membranes while accurately handling the given lipid ratios, and it has been broadened to handle the creation of systems without a membrane component. It was designed to be easy to use for a typical user, but also highly customizable in case of more specific system requirements. The software can be run both within a Python script or as a terminal command-line, and it uses a single command to specify all the system requirements.

The code, documentation, tutorials and license are available at github.com/MikkelDA/COBY.

## Theory

### Code Organization

COBY is written in Python 3 and the commands can be used either within the Python script, or as a command line in the terminal window. The entire simulation system is specified in a single command call. The requested system elements are then built in a serial fashion (Figure S1), where individual elements are added in a predefined order.

### Membrane and Protein Treatment

COBY treats leaflets as semi-independent components of the membrane and as such builds membranes on a leaflet-by-leaflet basis. If a protein is placed in the proximity of a leaflet, each protein bead is checked for potential overlap with the 3D leaflet space (Figure S2). By considering the bead overlap with the leaflets, rather than the whole membranes, COBY can better accommodate irregularities in protein shape. For large membranes, COBY performs dynamic segmentation of the leaflets for faster processing (Figure S3).

COBY contains tools for creating membrane patches and artificial membrane pores. These can be made either by using predefined shapes or by defining polygon points in the *xy*-dimension. It also features a specialized argument to create stacked membrane systems with customizable compositions of each individual membrane and solvent space.

### Lipid Packing

Correct lipid packing is dependent on the user providing reasonable APL values for each leaflet. While APLs of homogeneous membranes can be based on the lipid type APL, complex asymmetric membranes benefit from a more careful APL prediction. We recommend building two symmetric membranes (each corresponding to the composition of one leaflet) and running short MD simulations until the APL values are reasonably equilibrated. These APLs can then be used as an input for building asymmetric complex membranes.

The lipid packing procedure starts with determining an absolute number of lipids in leaflets, followed by an initial lipid placement, and finally the optimization of lipid positions. The lipid occupancy area (*A*_free_) is calculated by subtracting the area occupied by proteins or artificial pores from the total leaflet area. *A*_free_ is then used to calculate the highest allowed number of lipids (*N*_COBY,max_) from the specified area per lipid (APL). The result is then rounded to the nearest integer:

1 $$N_{COBY,\max}=\left[\frac{A_{free}}{APL}\right]$$

*N*_COBY,max_ is then allocated across the requested lipid types, taking their internal ratios into account, with the number of each lipid type being rounded down to the nearest integer. The resulting sum of lipids is considered the minimal allowed number of lipids (*N*_COBY,min_) in the leaflet,

2 $$N_{COBY,\min}=\overset{N_{\mathrm{types}}}{\underset{i=1}{\sum }}\left\lfloor \frac{w_{i}}{\overset{N_{\mathrm{types}}}{\underset{j=1}{\sum }}w_{j}}\cdot N_{COBY,\max}\right\rfloor$$

where *N*_types_ is the number of lipid types, *w* is the interlipid ratio of a given lipid, and *i* and *j* are the indices of the lipid types. The default algorithm first adds *N*_COBY,min_ lipids; then, *N*_COBY,max_ is reached by iteratively adding lipids of the most underrepresented type relative to the requested ratio. Other optimization algorithms with different prioritisation schemes are also available in COBY.

In the lipid placement step, all lipids are represented as circles in the *xy* plane and with a diameter that corresponds to its lipid type. The lipid types are divided into groups based on size, with the larger lipids being semirandomly placed on a 2D-grid with spacing adjusted for lipid sizes. Then, the smaller lipids are placed on the *y*-directional lines within the nonoccupied area of the leaflet (Figure S2f-h). The number of lines and the corresponding number of lipids on the line are dynamically optimized until all requested lipids are placed in the leaflet.

Following the initial lipid placement, any overlaps between the circles are resolved by using a distance-based optimization algorithm (Figure S2h–i). The lipids are exerting a pushing ”force” between each lipid pair within a cutoff distance, with the direction of the force informed by the vector between the two lipids, and the magnitude by the interlipid distance, optimization step (as the force is linearly scaled with each step), and the lipid size. (for details, see SI). The pushing is also applied from the edges of the membrane patch. Optimisation is completed when the maximum force on any lipid is smaller than a set tolerance value, or when the maximum number of steps is reached.

### Solvation

Solvation steps are independent of the solvent molecule of choice, and COBY can use any molecule as a solvent, as long as its structure and the molarity information are provided.

Solvation and flooding steps are performed in a 3D box space, where the box is rasterised, with the grid resolution determined from the length of the largest solvent molecule. The grid points that overlap with other system beads or the hydrophobic volume of a membrane are marked as unavailable for solvent insertion. Based on a given concentration, the total number of solvent particles is calculated using only the solvent-occupied volume.

Solvent neutralization is carried out in two steps, first by adding the desired concentration of ions, after which the solvation box is neutralized. COBY offers three neutralization algorithms, where (i) counterions are added, (ii) co-ions are removed to reach neutralization, or where (iii) the addition of counterions and removal of co-ions is combined. In mixed solvent cases, the intersolvent ratios can be interpreted either as ratios based on the CG bead numbers, or as ratios that take into consideration the underlying atom-to-bead mapping.

### Topology Handling

COBY reads topology files and uses them to obtain charge information for imported molecules. Topologies are unambiguously linked to the imported molecules by using the names specified in the [ moleculetype ] segment of the GROMACS topology file. The #include statements within the topology file are recursively processed, and the output topology file is updated with the specifications of the built systems and can directly be used in the simulation preprocessing steps in GROMACS.

### Parameter Libraries, Molecule Building, and Import

Molecular structures are organized in parameter libraries. Multiple libraries of the same type can coexist, and can be used to differentiate between multiple implementations of the same molecule. The library contents can be accessed interactively using the COBY.Library command. This feature is particularly interesting in a development context, where molecule parameters can be sourced from different development libraries.

Inspired by *insane*,^18^ we designed COBY so it can use internal fragment libraries to build the structures of molecules of interest (e.g., lipids with specific head, linker, and tail combinations). An existing set of fragments is provided as an in-built library, but the fragments can also be imported. The molecules are dynamically built by joining the building blocks at the specific attachment points. Depending on the type, lipids can contain a custom number of tails, which allows the user to import their own complex lipid components and use them to create custom lipids.

Molecules can be imported or created directly in the system-building command, or in a separate COBY.Crafter command call, which only outputs the structure of a requested molecule. By having an option to skip the system-building step, COBY can be used to straightforwardly provide structure files of the desired lipids, which is a routine requirement in the force field development process.

## Methods

### System Showcase

Eleven systems have been created to showcase the flexibility and versatility of COBY. The code used to create the systems can be found in the Github repository under the Tutorial section (https://github.com/MikkelDA/COBY), and the system details are listed in Table S1.

## Code accuracy comparison

### Total Number of Lipids

We compared the accuracy of COBY and *insane* algorithms of reproducing the *N*_ideal_ by monitoring the deviation from the ideal of the number of lipids within a leaflet (Figure 1a) or the lipid ratios between the leaflets (Figure 1b) as a function of membrane size. The input for both programs was the membrane size in *xy* dimension and APL.

3 $$N_{ideal}=\frac{A_{free}}{APL}$$

**Figure 1:**
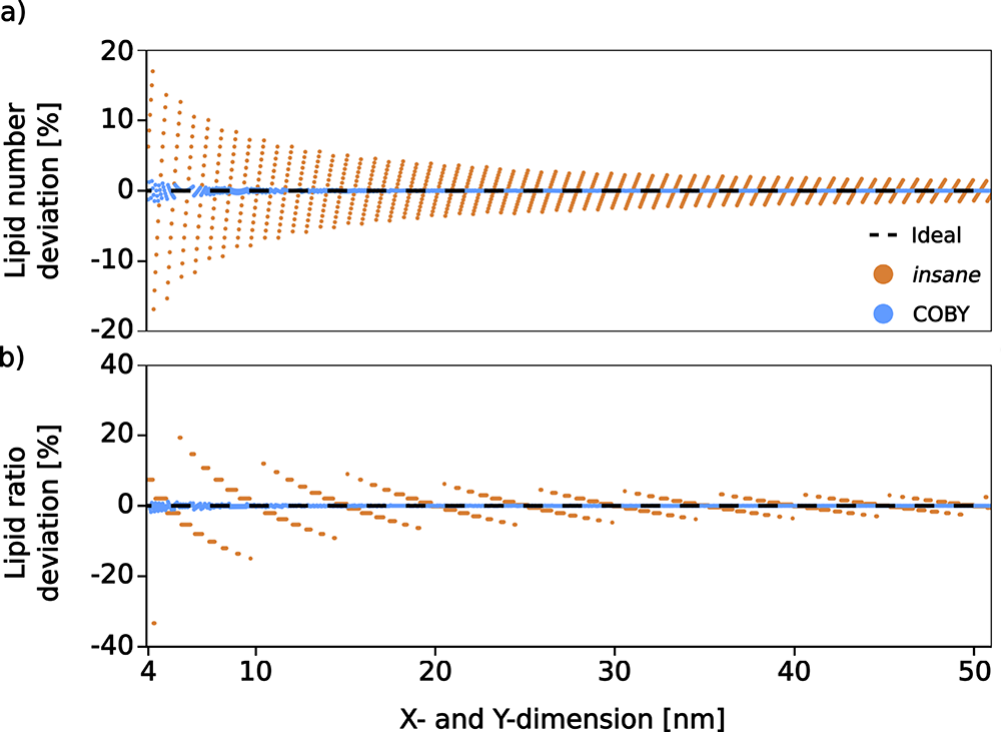
Comparison of accuracy in the membrane building step. The deviation of the number of lipids from the ideal values for COBY (in blue) and *insane* (in orange). Ideal deviation (always zero) is shown as a black dashed line. (a) The deviation of the number of lipids per leaflet compared to an ideal number (*N*_software_/*N*_ideal_) as a function of membrane size. (b) The deviation of interleaflet lipid ratios in asymmetric membranes (*N*_upper,software_:*N*_lower,software_/*N*_upper,ideal_:*N*_lower,ideal_) as a function of membrane size.

The constraints of the build—namely, that (i) the system is built with an integer number of lipids (ii) placed within a user-defined leaflet area and (iii) with consideration of a given APL—necessitate a compromise between the given properties so that the resulting deviation (*N*_software_/*N*_ideal_) is minimal.

The number of lipids inserted by *insane* in a leaflet (*N*_*insane*_) relies on two rounding operations and is calculated by

4 $$N_{insane}=\left[\frac{x}{\sqrt{APL}}\right]\cdot \left[\frac{y}{\sqrt{APL}}\right]$$

where *x* and *y* are the side lengths of a given membrane. The number of lipids inserted by COBY (*N*_COBY,max_) is calculated by eq 1.

The comparison of software accuracy based on the deviation from the requested lipid ratios was based on concurrent command calls to COBY and *insane* with an increasing box size (between 4 and 51 nm and by 0.05 nm increments) and with both leaflets assigned APLs of 0.6 nm^2^. We also assessed the deviation of interleaflet lipid ratios relevant for the asymmetric membranes (APL_upper_ = 0.60 nm^2^; APL_lower_ = 0.45 nm^2^), expressed as lipid ratio deviation (*N*_upper,software_: *N*_lower,software_/*N*_upper,ideal_: *N*_lower,ideal_).

### Speed Tests

Speed of COBY and *insane* was assessed by calculating the mean time to create a system over 5 attempts and over three system series (membrane-only, solvent-only, and with both membrane and solvent). The membranes were assigned POPC lipids (APL = 0.6 nm^2^), and the aqueous solvent contained 0.15 M NaCl. The *z* box dimensions were kept constant at 10 nm, while the *x* and *y* box size lengths were serially increased from 4 to 51 nm.

### Molecular Dynamics Simulations

All shown examples (Figure 2) have been minimized, equilibrated and simulated for a short period to confirm that the systems perform well in simulations without exhibiting any major instabilities. The details of the simulation procedure are available in SI.

**Figure 2:**
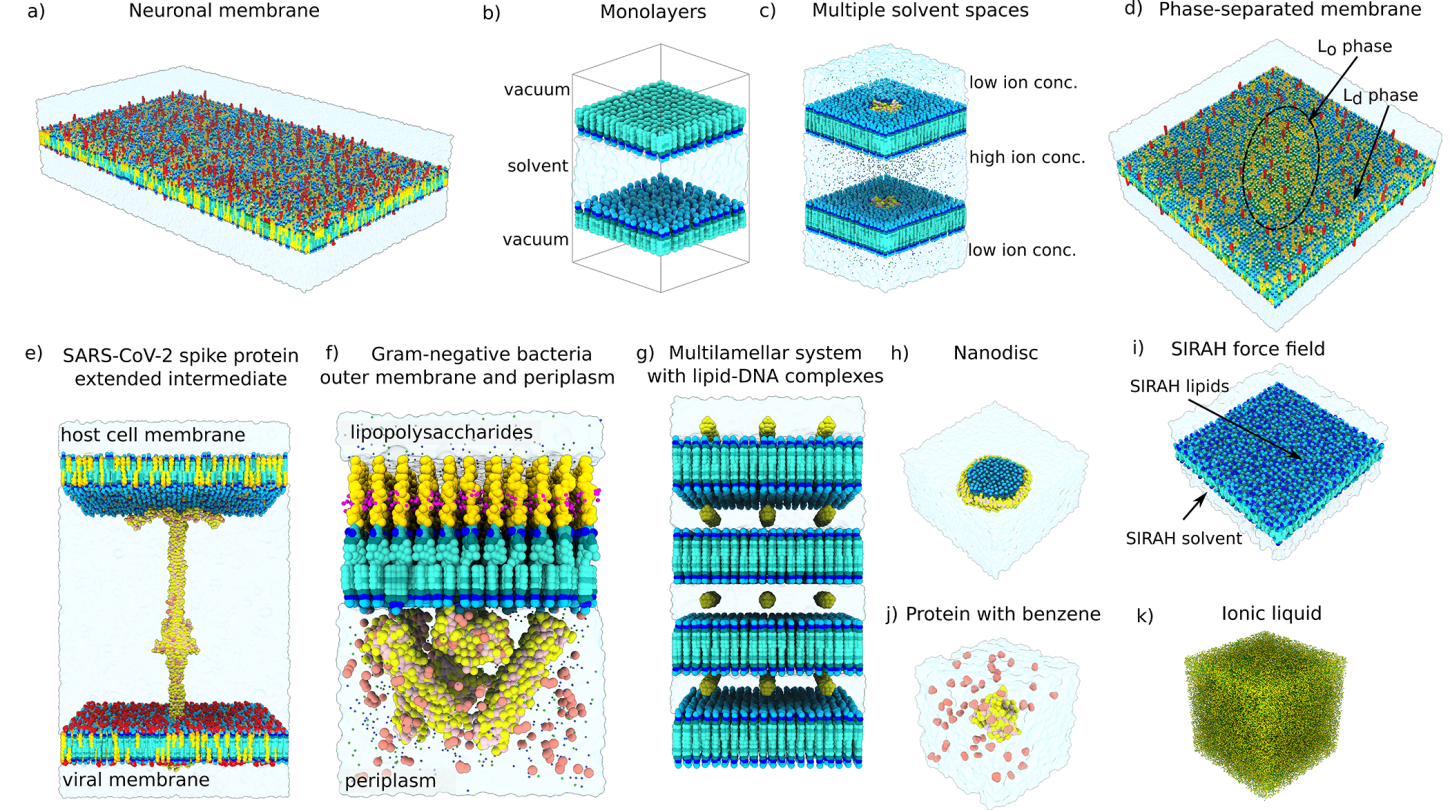
Showcase of systems built with COBY and using a single command-line call. (a) Complex asymmetric neuronal membrane consisting of 58 different lipid types using Martini 2 mapping scheme. Recreated from Ingólfsson et al.^22^ (b) Monolayer simulation setup. (c) Voltage-gated potassium channels (PDB: 3F5W)^23^ with modeled ion gradients contributing to the resting membrane potential.^24^ (d) A phase-separated giant unilamellar vesicle membrane with an ellipsoid liquid-ordered (L_o_) phase patch and a surrounding liquid-disordered (L_d_) membrane. Based on Hammond et al.^25^ (e) Extended intermediate of a SARS-CoV-2 spike protein^26^ spanning the viral^27^ and host cell membrane.^22^ (f) Complex outer membrane and periplasm of Gram-negative bacteria containing an asymmetric bilayer, four types of periplasmic proteins, and four types of solutes: spermidine, putrescine, glycerol and urea. Based on Pedebos et al.^28^ (g) Multilamellar lipid–DNA complexes used as artificial transfection agents, comprised of four vertically stacked membranes with dsDNA molecules occupying each intermembrane space.^29^ (h) A nanodisc in solvent.^30^ (i) Implementation of SIRAH molecules in COBY by importing solvent, ions, and lipids from the SIRAH force field.^31^ (j) A benzene flooding setup surrounding a human K-Ras protein (PDB: 4OBE).^32^ (k) An ionic liquid containing four types of cations and a corresponding number of tetrafluoroborate anions.

## Results

### Accuracy of Membrane Builds

The constraints of the membrane building procedure—the need to accommodate APL, lipid ratios, and a membrane patch size with an integer number of lipids—inherently lead to an error within one or more of the requested values describing the membrane properties. COBY was designed to minimize the build errors with a protocol described in the Theory section.

We examined the effect of membrane patch size on the deviation from the ideal number of inserted lipids, as dictated by the leaflet APL. We compared the performance of COBY with a commonly used CG membrane building software, *insane*. The deviation from the ideal number of lipids was appreciably higher for the membranes built by *insane* than it was for the membranes generated in COBY (Figure 1a). The deviation for *insane* membranes was especially large in small lipid patches and it tangentially reduced with the membrane size, but remained above 2% even in the patches that are larger than 50 nm × 50 nm. On the other hand, the deviation from the ideal lipid number in the membranes built by COBY dropped below 1% for the 6 × 6 nm^2^ membrane patch and remained under that value for all larger membranes.

We also assessed how the two algorithms compare in terms of preserving lipid ratios between the two leaflets in cases where the requested leaflet APLs are not the same (Figure 1b). While COBY effectively minimized the errors with only slight deviations present for the smaller membrane patches, *insane* deviations were appreciable even in larger membranes.

These differences in accuracy are due to the architecture of the underlying algorithms. COBY’s algorithm ensures that the number of lipids is as close to *N*_ideal_ as possible, and the variations are mainly confined within the decimal values. As the number of inserted lipids is an integer, these deviations are only observable in very small membrane patches. *insane* is comparatively more sensitive to the membrane size effects, as it assigns the number of lipids within a strictly defined 2D-grid informed by the *x*- and *y*-axis patch dimensions, and with an increasing patch size, adds whole rows and columns of lipids at one time, producing larger deviations from the ideal value.

An additional handling error specific to *insane* appears when handling intraleaflet lipid ratios, as the integer number of lipids is achieved by using a rounding down operation. This artifact is not present in COBY.

COBY is inferior to *insane* when it comes to speed (Figure S4). In particular, solvent insertion in COBY is slower than in *insane*, primarily because COBY takes into consideration free volume and solvent molarity in order to calculate the number of solvent molecules. In comparison, *insane* creates a simple 3D grid with spacing that roughly corresponds to the density of regular water beads. The advantage of COBY’s approach is that solvation is not restricted to water—instead, any solvent molecule can be used to solvate the system, provided solvent-specific molarity. We deemed this trade-off between accuracy and versatility vs speed a necessity, particularly when considering that even under these constrains, the creation of the larger systems (Figure 2a,d,e,f) took under a minute on an office workstation.

### Versatility of the Built Systems

The increase in system complexity has been a trend in the modeling community as a way to better reflect the native biological environment.^33^ Commonly, the system-building process is being carried out by nonstandardized procedures based on manual ”system-stitching”. We structured COBY in a way that all the elements of the system can be specified in a single command call, meaning that the program can optimize the building procedure with regards to the requested components. COBY uniquely offers considerations of molarity when using nonaqueous solvents, functionalities to build stacked membranes, phase-separated membranes, and multiple solvent spaces, all of which can be handled in combination within the same command call. These, in effect, can produce a great variety of reproducible high-complexity systems in a single step (Figure 2). Following is a brief overview of the diverse COBY functions that were used to create the models; for a more comprehensive overview of the systems’ biological relevance, code syntax, relevant considerations and limitations, the reader is referred to SI.

COBY can create of a broad spectrum of flat membrane-based systems: bilayers (Figure 2a,d,e) and monolayers (Figure 2b) of any given complexity, phase-separated membranes (Figure 2d), stacked membranes (Figure 2c,g), or membrane patches and pores of various shapes (Figure 2d,h).

Besides the membrane, COBY also handles placement of other system elements: proteins, solutes, and solvents. The system built by COBY can contain any number of proteins (Figure 2c,e,f) that can either be membrane-inserted or soluble (Figure 2j). Solvation can be controlled by the user to be either complete or partial (Figure 2b,c), and the solvents do not necessarily need to be aqueous, but instead use any other user-defined solvents (Figure 2k). Solute-flooding can also be performed in COBY (Figure 2f,j).

The package includes developer-friendly features, such as easy handling of multiple parameter libraries, importing lipid structures (Figure 2f,i) that can be used in the membrane building step, building molecules from fragments (Figure 2f), and importing solute molecules for the purposes of flooding (Figure 2f,j). Finally, while COBY defaults to ”best practice” calculation methods in order to build the desired system, it also offers a set of alternative algorithms that can be used instead (i.e., interleaflet lipid optimization options, multiple approaches to system neutralization and salting, and handling of mixed solvent ratios).

## Discussion

We created COBY, an open-source software for building CG systems with a primary focus on the accuracy of the building procedure, followed by the versatility of the produced output. The code has been designed to create systems with Martini CG models; however, the underlying code infrastructure can also be expanded to include other CG models (e.g., SIRAH,^31^ as shown in Figure 2i).

COBY demonstrates a greatly improved accuracy in system building, employing error minimization algorithms, which produce systems that reflect the properties requested by the user. Otherwise, a system-building software could create errors that are difficult to predict, as the deviations from the ideal are neither well documented nor linear. This also affects the replicability of simulation studies, as unassuming changes in the starting conditions (e.g., simulation box size) can lead to unexpected errors in the built systems (incorrect APL values of the membrane). The improvements implemented in COBY allow for an accurate creation of multicomponent systems, and alleviate the burden on the user to check, detect, and correct the errors generated by the software.

We designed COBY with complex systems in mind and, as such, the software is able to handle a great variety of user requests within a single command line. This is crucial in high-throughput protocols that use MD simulations, as they rely on automated procedures to execute the entire pipeline,^34^ and should ideally not be restricted by the limited complexity of the available system-building methods. The automated procedure also provides a straightforward approach to reliably replicate the simulation experiment. Note that the package is best suited to build the systems in 100 nm-size range, as bigger system sizes exponentially increase the computation time (Figure S4) or have high memory requirements, especially concerning the solvent creation step. As COBY is designed to only handle flat membranes, the curved membrane systems could be created with the TS2CG software instead.^17^

Force field development is reliant upon extensive testing of the new parameters in a streamlined manner. COBY parameter libraries are organized in a way that makes it possible to use multiple libraries within the same command call, allowing the user to mix and match the parameters of choice. Users can also create new libraries either by directly importing them, or by importing individual molecules from structure files and optional topology files. In addition, a big portion of lipids can be built in COBY from fragments available from the in-built libraries.

COBY is a tool that is meant to have a broad user base, as the simple systems can be built with a very few mandatory arguments—namely, box determining the size of the system, and at least one other element (e.g., membrane, solvent, protein, or solute). At the same time, it offers a great deal of flexibility to the experienced user wishing to employ it for complex system building or systematic parametrization procedures. Possible implementation of COBY within a graphical user interface environment, such as MAD,^35^ could further simplify the system-building procedure for many users.

## Supplementary Material



## Data Availability

The open-source code, basic and advanced tutorials, code for creating showcased systems, cheat sheet, and detailed documentation are hosted on the project page at https://github.com/MikkelDA/COBY.

## Notes

Figures illustrating COBY workflow, membrane creation process, membrane segmentation procedure, speed test comparison, a table detailing the showcase systems (for Figure 2), a description of the pushing force used in the optimization algorithm, the MD simulation protocol, and additional information for showcased systems (PDF)
