# Supplementary material for: Creating Coarse-Grained Systems with COBY: Toward Higher Accuracy of Complex Biological Systems
Source: J Chem Inf Model. 2025 May 12;65(10):4760–6. doi: 10.1021/acs.jcim.5c00069 (PMC12117566; doi:10.1021/acs.jcim.5c00069)
Supplement: Supplementary file 1 [file ci5c00069_si_001.pdf]

Creating Coarse-Grained Systems with COBY: Towards Higher Accuracy of Complex Biological Systems

Mikkel D. Andreasen,[†] Paulo C. T. Souza,[‡,¶] Birgit Schiøtt,[†] and Lorena Zuzic[*,†]

†*Department of Chemistry, Aarhus University, Langelandsgade 140, 8000, Aarhus C, Denmark*

‡*Laboratoire de Biologie et Modélisation de la Cellule, CNRS, UMR 5239, Inserm, U1293, Universite Claude Bernard Lyon 1, Ecole Normale Supérieure de Lyon, 46 allée d'Italie, 69364, Lyon, France*

¶*Centre Blaise Pascal de Simulation et de Modélisation Numérique, Ecole Normale Supérieure de Lyon, 46 allée d'Italie, 69364, Lyon, France*

E-mail: lorena.zuzic@chem.au.dk

Supplementary Information

List of Figures

S1 COBY workflow. 3

S2 Membrane leaflet creation process in COBY. 4

S3 Automatic membrane segmentation. 5

S4 Speed comparison between COBY and *insane*. 7

List of Tables

S1 Overview of the showcased systems. 6

Contents

1 Pushing force algorithm 8

2 MD simulation protocols 9

3 Building challenges and biological significance of showcase systems 10

References 19

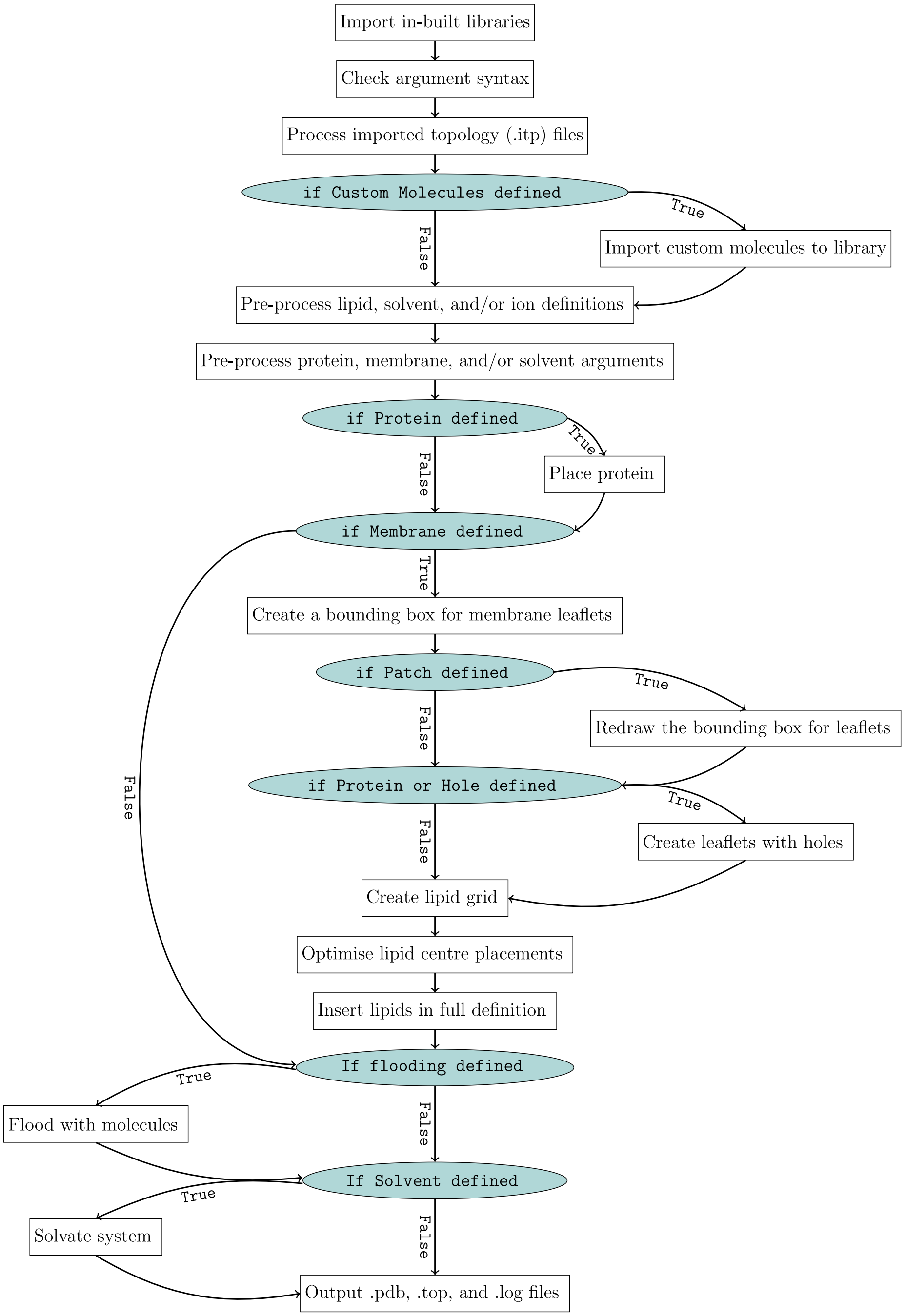

Figure S1: **COBY workflow.** The simplified code workflow shows the if-statements in teal circles, Boolean evaluations next to arrows, and the procedure steps in white boxes.

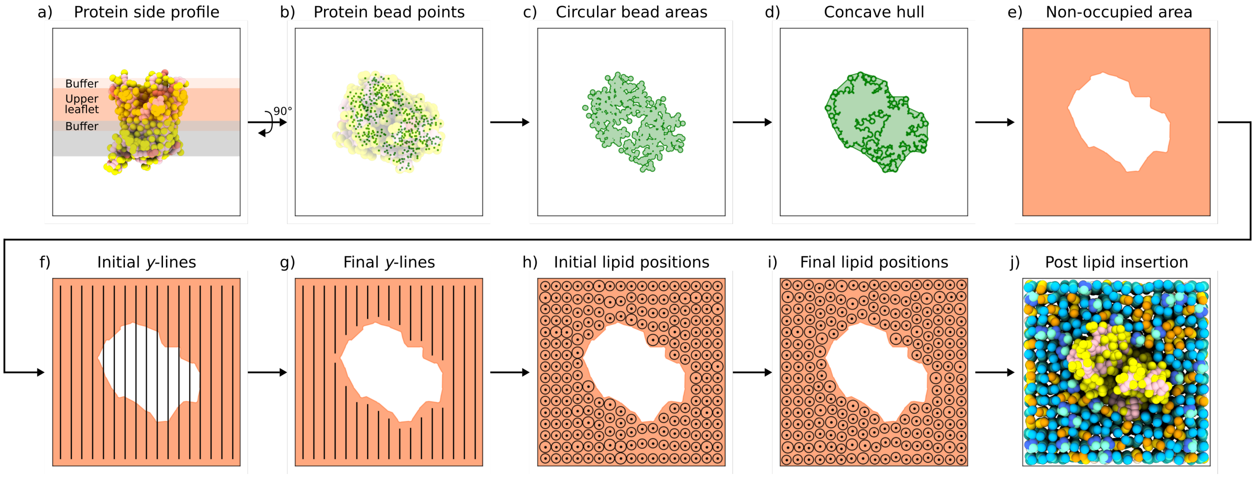

Figure S2: **Membrane leaflet creation process in COBY.** **(a)** Protein beads that are occupying leaflet and buffer spaces are **(b)** flattened onto a 2D surface as points. **(c)** The bead points are converted to circles using the Python module `shapely` and are merged if they are overlapping. **(d)** A concave hull is calculated from the surface points using the Python module `alphashape`, with an α value calculated from the diameter of the lipids present in the leaflet and an additional buffer distance. **(e)** A negative of the occupied space is used for lipid insertion. **(f)** A series of y-lines describing the possible placement of lipids is created. **(g)** The line segments that traverse the occupied areas of the leaflet are removed. The resulting line segments are then assigned a number of lipids that can be fit along the y-line. If the total number of lipids in a leaflet is too small, then the density of the y-lines or the density of the aligned lipids is increased, and the process is repeated. **(h)** All lipids are represented as circles with diameters corresponding to the specific lipid type. Lipid circles are inserted onto the grid in a random order, and **(i)** any overlaps are prevented by an internal optimisation algorithm. Finally, **(j)** the lipid and protein beads are placed in the 3D system.

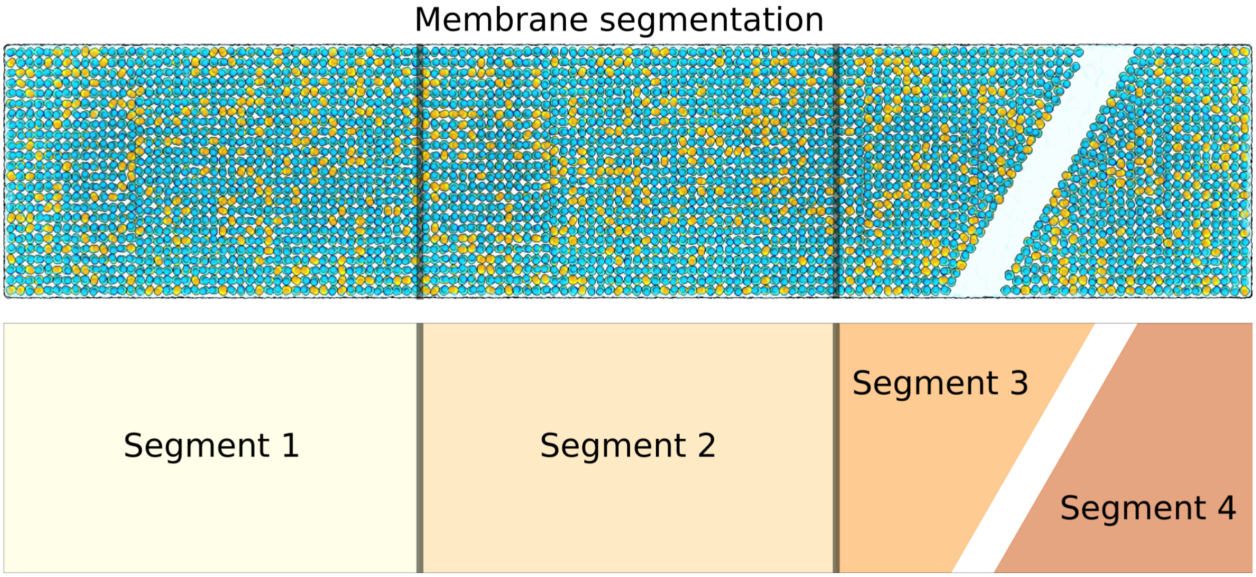

Figure S3: **Automatic membrane segmentation.** An example of how a membrane is dynamically split into multiple segments, based on size and connectivity to improve performance and accuracy. A larger membrane patch is split into three parts (segments 1-3), while the smaller patch is treated as one unit (segment 4).

Table S1: Overview of the built systems showcased in Fig. 2 with membrane, protein, and solvent compositions.

**Figure panel**	**Membrane composition**	**Protein**	**Solvent**	**Reference**
**a)** Complex neuronal membrane	Complex neuronal membrane consisting of 58 lipid types (B58) [1]	-	Water and 0.15 M NaCl	Ingólfsson et al. [1]
**b)** Monolayers on the vacuum-solvent interface	Two monolayers: POPC	-	Bridging the monolayers: water and 0.15 M NaCl;<br>Across PBC: vacuum	-
**c)** Voltage-gated channels with modelled transmembrane potential	Two stacked membranes: POPC[i]	Two KcsA Potassium channels (PDB: 3F5W) [2]	Between the membranes: Water and 0.4 M NaCl;<br>Across PBC: Water and 0.15 M NaCl	Kutzner et al. [3]
**d)** Phase-separated membrane modelled after a giant unilamellar vesicle	Central patch: liquid-ordered phase (composition a[4]);<br>Surrounding membrane: liquid-disordered phase (composition b[4])	-	Water and 0.15 M NaCl	Hammond et al. [4]
**e)** Extended intermediate of the SARS-CoV-2 spike protein spanning the two membranes	Host cell membrane: an average membrane consisting of 8 lipid types (A8); [1]<br>Viral membrane: SARS-CoV-2 envelope membrane consisting of 7 main lipid types [5]	Extended intermediate model of the SARS-CoV-2 spike protein [6]	Water and 0.15 M NaCl	Su et al. [6]<br>Ingólfsson et al. [1]<br>Saud et al. [5]
**f)** Gram-negative bacteria outer membrane and periplasm	Bottom (outer) leaflet: POPE[ii], POPG[iii] and CDL0[iv];<br>Top (inner) leaflet: LPS[v].	four BLP (PDB: 1EQ7), one LolB (PDB: 1WLM), one Pal (PDB: 2W8B) and one OmpA (Zenodo: 1636577). [7]	Water, 0.2 M NaCl, 0.02 M spermidine, 0.03 M putrescine, 0.035 M glycerol, 0.03 M urea and 10 Mg^{2+} per RAMP lipid	Pedebos et al. [7]
**g)** Multilamellar systems of DNA complexed with membranes	All membranes: DOPE[vi]	12 24-base-pair dsDNA. [8] Obtained from the Martini website.	Water and 0.15 M NaCl	Corsi et al. [9]
**h)** Nanodisc in solvent	DMPC[vii]	Two apolipoproteins AI (PDB: 6CLZ) [10]	Water and 0.15 M NaCl	Marcink et al. [10]
**i)** Membrane and solvent using SIRAH force field mapping	SIRAH DMPC[vii]	-	SIRAH water WT4 and 0.15 M NaCl	Klein et al. [11]
**j)** Benzene flooding with a soluble protein	-	K-Ras (PDB: 4OBE) [12]	Water, 0.15 M NaCl and 0.1 M benzene	Hunter et al. [12]
**k)** Ionic liquid	-	-	Cations: EMIM[viii], BMIM[ix], OMIM[x] and DMIM[xi];<br>anions: BF4[xii]	-

[i] POPC: 1-palmitoyl-2-oleoylphosphatidylcholine
[ii] POPE: 1-palmitoyl-2-oleoylphosphatidylethanolamine
[iii] POPG: 1-palmitoyl-2-oleoylphosphatidylglycerol
[iv] CDL0: cardiolipin
[v] LPS: lipopolysaccharide
[vi] DOPE: 1,2-dioleoyl-phosphatidylethanolamine
[vii] DMPC: 1,2-dimyristoylphosphatidylcholine
[viii] EMIM: 1-ethyl-3-methylimidazolium
[ix] BMIM: 1-butyl-3-methylimidazolium
[x] OMIM: 1-ethyl-3-octylimidazolium
[xi] DMIM: 1-decyl-3-methylimidazolium
[xii] BF4: tetrafluoroborate

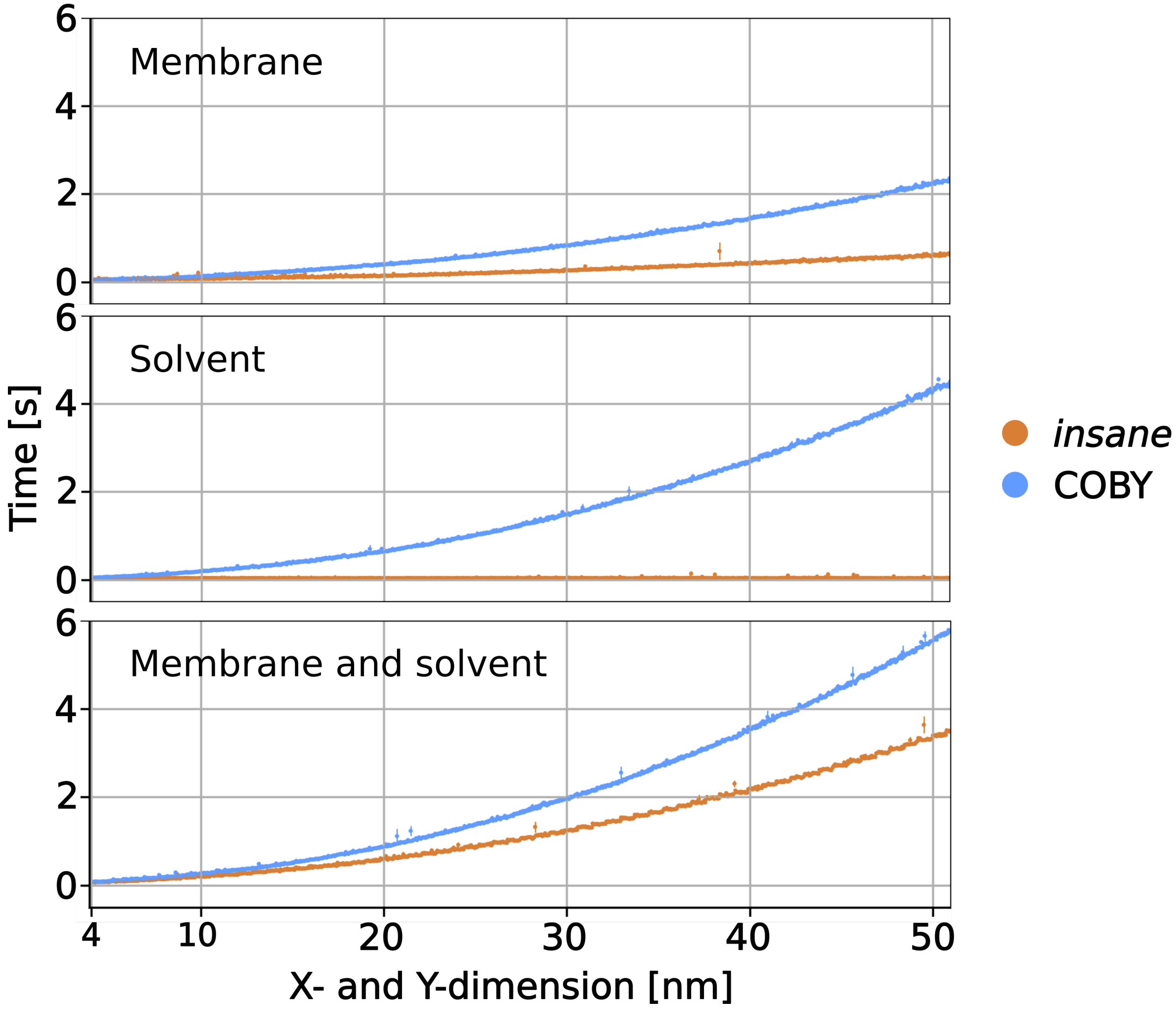

Figure S4: **Speed comparison between COBY and *insane*.** COBY performance is shown in blue, and *insane* in orange. The speed tests were performed on systems of an increasing size in the x and y direction, while the z-dimension was kept constant at 10 nm. Each data point was tested five times and is shown as the mean value with the standard error. The tests were performed on systems containing only membrane, only solvent, or the combination of both. The membranes consisted of POPC lipids (APL $= 0.6$ nm^2), while the solvent contained water and 0.15 M NaCl. Following the best-use practice, COBY was run from a Jupyter notebook, and the speed tests did not include the time required to import packages (which takes additional 3-4 s, depending on the available hardware). *insane* was run from a terminal.

1 Pushing force algorithm

The "pushing forces" do not directly correspond to the physical forces; instead, the optimisation algorithm is based on a set of simple and empirically developed procedures shown in Eq. 1-8. Note that the pushing forces between the lipids are only calculated for lipids pairs where $\|\mathbf{v}_{ij}\| \leq d_{\mathrm{max}}$ (defined below).

For $\|\mathbf{v}_{ij}\| \leq d_{\mathrm{max}}$:

$$A_{\mathrm{empty}} = A_{\mathrm{leaflet}} - \sum_{l}^{n_{\mathrm{lipids}}} 4r_l^2 \tag{1}$$

where A_{leaflet} denotes the total area of the leaflet, and A_{empty} is calculated by subtracting the summed area of all lipids from A_{leaflet}. The lipid area is calculated by representing the lipids as squares.

$$d_{\mathrm{min}} = r_i + r_j \tag{2}$$

$$d_{\mathrm{max}} = d_{\mathrm{min}} \left(2 \cdot \frac{A_{\mathrm{empty}}}{A_{\mathrm{leaflet}}} + 1 \right) \tag{3}$$

Distances d_{min} and d_{max} are the lower and upper boundaries of the lipid distances, respectively.

$$k_{\mathrm{step}}(s) = \left(1 - \frac{s-1}{15} \right) \tag{4}$$

The $k_{\mathrm{step}}(s)$ is a damping function that progressively reduces the forces on the particles with each optimisation step s.

$$\vec{f}(\vec{\mathbf{v}}_{ij}, k_{\text{step}}(s)) = \vec{\mathbf{v}}_{ij}\left[(d_{\max} - \|\mathbf{v}_{ij}\|)\cdot k_{\text{step}}(s) + \frac{d_{\min} - \|\mathbf{v}_{ij}\|}{2}\cdot u\right]\begin{cases} u = 1, & \text{if } \|\mathbf{v}_{ij}\| \leq d_{\min} \\ u = 0, & \text{otherwise}\end{cases} \tag{5}$$

The 2D vector $\vec{\mathbf{v}}_{ij}$ connects the centres of lipids i and j, and the force $\vec{f}$ is calculated based on the vector magnitude $\|\mathbf{v}_{ij}\|$ (describing the distance between the two lipids), and the $k_{\text{step}}(s)$ function described above. Note that the second term, $\frac{d_{\min} - \|\mathbf{v}_{ij}\|}{2}$, is only applied if the two lipids are overlapping.

Finally, the force is modified by k_{size} for the smaller lipid. Let $r_i \leq r_j$:

$$k_{\text{size}}(r_i, r_j) = \frac{r_i}{r_j} \tag{6}$$

$$\vec{F}_i(\vec{f}, k_{\text{size}}) = k_{\text{size}} \cdot \vec{f} \tag{7}$$

$$\vec{F}_j(\vec{f}) = \vec{f} \tag{8}$$

The force magnitude will be scaled based on the distance between the lipid pair, the optimisation step, and the lipid size (with smaller lipids receiving a weaker push). The force orientation will correspond to $\vec{\mathbf{v}}_{ij}$ for one lipid, and to $-\vec{\mathbf{v}}_{ij}$ for the other lipid. The pushing force from the edge of the leaflet is calculated in an equivalent manner, with vector $\vec{\mathbf{v}}_{ie}$ describing the shortest path between the centre of the lipid i and the edge e.

2 MD simulation protocols

All systems were minimized for 2000 steps using the steepest-descent algorithm and then equilibrated for 20 ns in an *NPT* regime with proteins weakly position-restrained ($F_c = 20$

kJ mol^{-1} nm^{-2}). The systems were simulated for 100 ns with a 20 fs time step and using the v-rescale thermostat[13] at 310 K ($\tau_t = 1.0$) and c-rescale barostat[14] at 1 bar ($\tau_p = 4.0$ and compressibility at 3.0×10^{-4}) with the pressure-coupling scheme appropriate for the system type (semi-isotropic for systems with a membrane spanning the xy plane, isotropic for the systems without it). Van der Waals interactions were considered up to the cutoff of 1.1 nm and with a potential-shift Verlet scheme with the same cutoff; the reaction-field electrostatics were considered to the 1.1 nm cutoff ($\epsilon_r = 15$, $\epsilon_{\mathrm{rf}} = 0$). The pressure in the system containing SIRAH molecules was instead maintained using the Berendsen barostat.

3 Building challenges and biological significance of showcase systems

Figure 2 features eleven complex systems that are created using a single command call by combining a specific set of COBY arguments. The choice of systems was inspired by the practical need of such setups in biological or biotechnological contexts. For each case we list the relevance of the model, possible building challenges and associated solutions provided by COBY, building shortcuts, and code snippets showing the relevant syntax. Complete and reproducible system building procedures are available at github.com/MikkelDA/COBY.

Neuronal membrane

A common example of demanding systems built in CG resolution are complex membranes (Fig. 2a). In our example, we recreated a neuronal membrane model from Ingólfsson *et al.*[1] that contained 58 different lipid types. On top of being highly complex in terms of lipid species, the neuronal membrane was also asymmetric, which was reflected in different leaflet APL values. All lipid types were imported from a Martini 2 lipid library file. The entire membrane building procedure and solvation took under 30 seconds on an office workstation.

```
sysname = "NeuronalMembrane"
```

```
COBY.COBY(
    ### Box size
    box = [90, 50, 20], # [nm]

    ### Imports the Martini2 lipid library (library name: IngolfssonMembranes)
    import_library = "example_molecule_definitions/NeuronalPlasmaMembraneAverage_lipid_scaffolds.py",

    ### A single membrane is specified as a single string, but for visual clarity, the leaflets are written on separate lines.
    ### As there are 58 different lipid types, we omitted most of the lipid specifications (denoted by [...]).
    ### If subargument(s) are written before the leaflet designation, they apply to the whole membrane (in this case, params).
    ### If subargument(s) are written after the leaflet designation, they apply to the specific leaflet, up until:
    ###    a) the end of the string, or
    ###    b) a new leaflet subargument appears.
    membrane = [
        "params:IngolfssonMembranes leaflet:upper apl:0.460 lipid:DPPC:0.053 lipid:POPC:0.087 [...] \
        leaflet:lower apl:0.485 lipid:DPPC:0.030 lipid:POPC:0.049 [...]"
    ],

    ### Imports itp files for Martini 2 lipids and ions. Note that multiple itp files can be handled at the same time
    itp_input = [
        "include:toppar/NeuronalPlasmaMembraneBrain/martini_v2.2.itp",
        "include:toppar/NeuronalPlasmaMembraneBrain/martini_v2.0_brain_complex_b5-GMs_old.itp",
        "include:toppar/NeuronalPlasmaMembraneBrain/DPPX_Martini_v2.0_lipid.itp",
        "include:toppar/NeuronalPlasmaMembraneBrain/POPX_Martini_v2.0_lipid.itp",
        "include:toppar/NeuronalPlasmaMembraneBrain/martini_v2.0_ions.itp",
    ],

    ### Output files
    out_sys = sysname + ".pdb",
    out_top = sysname + ".top",
    out_log = sysname + ".log",

    ### Designates the system name that is written in structure and .top files
    sn = sysname,

    ### Omitted some arguments for clarity. For a full command, please see the Tutorial on Github.
    [...],
)
```

Monolayers

COBY can easily handle monolayers, as the code by default performs most of the membrane building steps in the leaflet subspace. A monolayer setup that is commonly used for MD simulations features two monolayers facing each other, a layer of solvent between them, and the vacuum region spanning the tails (Fig. 2b). The system was built in a single step by specifying the offset between the two monolayers, and the composition of the surrounding space (either an aqueous solution or vacuum).

```
COBY.COBY(
    ### Creates two separate monolayer membranes, each placed in the center of the x/y-plane.
    ### Separate membranes/monolayers are specified as separate strings within the membrane argument.
    membrane = [
        "type:mono_lower lipid:POPC center:0:0:5", # [nm]
        "type:mono_upper lipid:POPC center:0:0:-5", # [nm]
    ],

    ### default: water + 0.15 NaCl.
    ### The "zlength:10" subargument designates that the solvent box should only be 10 nm long along the z-axis.
    ### This results in the solvent only being placed between the monolayers.
    solvation = "default zlength:10",

    [...], # Other arguments
)
```

Multiple solvent spaces

Multilayered membrane systems can also be created more stringently by using a special `stacked_membranes` argument. Stacked membrane systems are often used for simulating transmembrane potential conditions, as they feature multiple isolated solvent spaces and solvents of different compositions. In this example, two voltage-gated ion channels — one in each membrane — were positioned across two solvent spaces, with the extracellular segments facing the high-ion concentration solvent, and the intracellular components oriented towards the low-ion concentration solvent (Fig. 2c). One of the proteins was rotated 180° around the x-axis in order to face the same solvent space.

```
COBY.COBY(
    ### Designates the box size without the z-dimension, as this will be determined from the stacked_membranes argument.
    box = [16, 16], # [nm]

    ### Line 1: general stacked_membranes properties (number of membranes, distance, distance type)
    ### Line 2: membrane properties (in this case, the same for both membranes, as specified by positions:1:2)
    ### Line 3: solvent 1 property (crossing the PBC)
    ### Line 4: solvent 2 property (between the two membranes)
    stacked_membranes = [
        "number:2 distance:10:10 distance_type:surface \
        membrane_argument:positions:1:2 apl:0.5 lipid:POPC \
        solvation_argument:positions:1 default \
        solvation_argument:positions:2 default salt_molarity:0.4"
    ]

    ### The system contains two proteins of the same type (one in each membrane)
    ### The protein consists of four chains, all present in the same .pdb input file.
    ### Each chain has its own moleculetype assignment in the .itp file
```

```
    protein = [
        "file:3f5w_martinized.pdb moleculetypes:3f5w_chainA:3f5w_chainB:3f5w_chainC:3f5w_chainD cen_method:axis cz:7",
        "file:3f5w_martinized.pdb moleculetypes:3f5w_chainA:3f5w_chainB:3f5w_chainC:3f5w_chainD cen_method:axis cz:-7 rx:180",
    ]

    ### Topology files
    itp_input = [
        "file:toppar/top_for_COBY.itp",
        ### Contains references to all 4 itp files for the protein, each containing 1 moleculetype
        "file:toppar/PotassiumChannel/3f5w_martinized.top",
    ],
    [...], # Other arguments
)
```

Phase-separated membrane

A phase-separated system was inspired by the experimental insights on the organisation of a giant unilamellar vesicle (Fig. 2d). The liquid-liquid phase-separation was modelled by creating an ellipsoid `patch` with a lipid composition conducive to liquid-ordered phase, while the surrounding membrane, containing a complimentary ellipsoid `hole`, was built with a lipid composition promoting the liquid-disordered phase organisation. In general, COBY can handle any 2D shape for the patch or pore creation, as the common shapes (circles, ellipses, rectangles) are complimented by the polygon function that can create shapes with an N number of vertices.

```
COBY.COBY(
    # The membrane with a hole and a membrane patch are treated as two separate membranes, and therefore are they placed in individual strings
    # ellipse arguments are the same for both membranes, thus overlapping the hole/patch placement
    membrane = [
        "params:IngolfssonMembranes lipid:DOPC:44.1 DOPG:4.9 CHOL:30 DPG1:2 hole:ellipse:xradius:8:yradius:16:cx:0:cy:0:rotate:45",
        "params:IngolfssonMembranes lipid:DOPC:9 lipid:DOPG:1 lipid:DPSM:53 lipid:CHOL:35 lipid:DPG1:2 \
              patch:ellipse:xradius:8:yradius:16:cx:0:cy:0:rotate:45",
    ],
    [...], # Other arguments
)
```

SARS-CoV-2 spike protein extended intermediate

Since the COVID-19 pandemic, computational modelling of viral machinery associated with viral entry, replication, or maturation has become an important component of research into

the mechanisms of viral infections. One feature of such models is a hybrid environment that involves both viral and host cell components, both of which are often included in the computational model. We built a system featuring an extended spike protein of SARS-CoV-2 spanning the viral and the host cell membrane (Fig. 2e). This extended intermediate is formed when the viral fusion peptides are inserted into the host cell membrane and preclude the viral-host membrane fusion event essential for successful viral entry into the cell. The system was built in a single COBY command-call by creating two complex membranes with different compositions using the `stacked_membranes` argument, and by positioning the extended intermediate so that it spans the solvent space between the two membranes. The system creation took around 10 seconds on an office workstation.

Gram-negative bacteria outer membrane and periplasm

Inherently, crowded biological systems contain many different lipids, proteins and solutes in a restricted space. COBY can insert any number of proteins, membranes and solute types, and perform solvation and flooding in restricted box volumes (Fig. 2f). We used COBY to create a system inspired by the model of the bacterial periplasm presented in Pedebos *et al.*[7] The system contained an asymmetric membrane with phospholipids and cardiolipins in the inner leaflet and lipopolysaccharides (LPS) in the outer leaflet. The phospholipids were imported from a Martini 2 lipid library using the `import_library` argument, the structure of the LPS was imported from a pdb file using the `molecule_import` argument, and the cardiolipin was built using the molecule fragment builder `molecule_builder` argument with the fragments having been imported from a fragment defintions file using the `import_library` argument. Magnesium ions have been inserted using the `solvation` argument inside the LPS leaflet. Four protein types were inserted: an outer membrane protein A (OmpA) monomer located in the centre containing a long membrane-penetrating portion, four Braun's lipoproteins (BLP) and one outer membrane lipoprotein receptor (LolB) in the periplasm, and one outer membrane lipoprotein (Pal) located in the periplasm near the membrane. The periplasm was

also flooded with 0.02 M spermidine, 0.03 M putrescine, 0.035 M glycerol, 0.03 M potassium, and 0.2 M chloride ions. Parameters for the solutes were obtained using auto_martini.[15]

When generating a system of this size of complexity, there are certain limitations to what can be created with COBY software. Specifically, creating a peptidoglycan wall is unsupported by the package, and was thus excluded from the model. This problem can be circumvented by importing a pre-made peptidoglycan wall. This "stitching" approach, however, is in opposition to the overall spirit of the package, and was therefore omitted from the building procedure.

```
COBY.COBY(
    ### solvate_hydrophobic_volume combined with solvation inserts only Mg2+ ions in the upper leaflet
    membrane = [
        "cz:2 \
        leaflet:lower params:IngolfssonMembranes lipid:POPE:90 lipid:POPG:5 lipid:CDL0:5:params:MOLBUILDER apl:0.644 \
        leaflet:upper params:IMPORTED lipid:RAMP solvate_hydrophobic_volume:True apl:1.660"
    ],

    ### Building CDL0 using the molecule_builder by importing fragments with import_library
    molecule_builder = [
        "moltype:m2_cardiolipin tail11:CCDCC tail21:CCDCC tail12:CCDCC tail22:CCDCC name:CDL0 params:MOLBUILDER",
    ],

    solvation = [
        ### Spermidine - Doing this one first as it is the largest one
        "solvent:name:SPER:params:IMPORTED solv_molarity:0.020 center:0:0:-5 zlength:10",
        ### Putrescine - Doing this one second as it is the second largest one
        "solvent:name:PUT:params:IMPORTED solv_molarity:0.030 center:0:0:-5 zlength:10",
        ### Glycerol
        "solvent:name:GLYL:params:IMPORTED solv_molarity:0.035 center:0:0:-5 zlength:10"
        ### Urea
        "solvent:name:UREA:params:IMPORTED solv_molarity:0.030 center:0:0:-5 zlength:10"

        ### Mg2+: inserted inside the outer leaflet
            ### Sets some solvent placement values to ease placement between lipids
            ### Allows placement of molecules inside leaflets that are also designated with "solvate_hydrophobic_volume:True"

        "solvent:MG:params:IMPORTED solv_per_lipid:10 solvate_hydrophobic_volume:True center:0:0:-5 zlength:10 \
            kick:0 gridres:0.05 buffer:0.001 protein_extra_buffer:1",

        ### Solvent and ions; 1% of water is represented as "WF" / "anti-freeze water"
        "params:M2 solv:W:99 solv:WF:1 pos:NA neg:CL salt_molarity:0.2",
    ],

    ### Importing RAMP lipid and osmolytes
    molecule_import = [
        "file:example_lipids/M2_RAMP_RaLPS.pdb moleculetype:RAMP params:IMPORTED scale:x:0.5 scale:y:0.5",
        "file:example_solutes/M2_BacterialMembrane/GLYL.gro moleculetype:GLYL params:IMPORTED",
        [...],
```

```
    ],

    ### Imports the Martini2 lipid definition libraries
    import_library = [
        ### Molecule definitions for various Martini2 lipids
        "example_molecule_definitions/NeuronalPlasmaMembraneAverage_lipid_scaffolds.py",

        ### Fragment definitions for Martini2 cardiolipins
        "example_molecule_definitions/fragment_defs_cardiolipins_m2.py",
    ],

    [...], # Other arguments
)
```

Multilamellar system with lipid-DNA complexes

Computational modelling approaches can also be applied to nanostructures that usually cannot be found in biological systems. Multilamellar systems, such as cationic lipid–DNA complexes, are used as artificial transfection agents for efficient DNA delivery into the cells. On a molecular level, they feature multiple layers of DNA sandwiched between cationic membranes (Fig. 2g). We created the complex by utilising the `stacked_membranes` argument to define the placement of the membranes and the size of the inter-membrane spaces, while the DNA molecules were placed in each solvent space using the `protein` argument (which is in practice agnostic about the type of the specified biomolecule). This system was inspired by Corsi *et al.*[9] and modelled with the Martini 2 mapping scheme.

Nanodisc

Another example of sythetic constructs are nanodiscs, which can be used for the *in vitro* studies of membrane proteins. COBY was used to create a nanodisc in solvent (Fig. 2h), where a specialised membrane subargument was used to specify lipid placement within the borders of the apolipoprotein, as opposed to the default lipid placement around the protein. The code also handled the solvation of the remainder of the box unoccupied by the nanodisc.

```
COBY.COBY(
    ### inside_protein specifies if the membrane can be placed within the protein (as is the case with nanodiscs).
    ### solvate_hole specifies if the rest of the membrane plane that does not contain lipids should be solvated
    membrane = [
```

```
        "lipid:DLPC inside_protein:True solvate_hole:True",
    ],

    ### membrane_border specifies that this protein should describe the membrane border (complimentary with inside_protein)
    protein = [
        "file:example_proteins/6clz_nanodisc_martinized.pdb moleculetypes:nanodisc_ring1:nanodisc_ring2 membrane_border:True",
    ],
    [...], # Other commands
)
```

Protein with benzene

COBY can also perform flooding of systems with user-imported solutes, exemplified by the benzene flooding setup of the K-Ras protein (Fig. 2i). The benzene mapping setup can be used in MD simulations for detecting cryptic pockets,[16] and the approach is therefore particularly interesting for the proteins that are both medically relevant and difficult to drug. Both K-Ras and benzene were imported from their respective structure files, and benzene molecules were added to the random positions and orientations in the solvent in a user-specified concentration, while ensuring no overlaps with the protein. COBY correctly handled solvent and ion concentrations even after the addition of solute molecules, as it considered only the unoccupied volume for calculating the required number of solvent and ion particles.

```
COBY.COBY(
    ### KRAS protein containing 1 Mg ion.
    protein = [
        "file:example_proteins/4obe_martinized_withMG.pdb moleculetypes:4obe:MG"
    ],

    ### The system is solvated with benzene in 0.1 M concentration and with water + 0.15 M NaCl (default)
    solvation = [
        "solvent:BENZ solv_molarity:0.1",
        "default",
    ],

    ### Importing benzene structure file
    molecule_import = [
        "file:example_solutes/M3_small_molecules/BENZENE/BENZ.gro moleculetype:BENZ",
    ],

    ### Importing itp files: for Martini 3, KRAS protein, Mg2+, and benzene
    itp_input = [
        "file:toppar/top_for_COBY.itp",
```

```
        "include:toppar/KRAS/4obe_martinized.itp",
        "include:toppar/extra_ions/martini3_Mg_ion.itp",
        "include:toppar/solutes/M3_small_molecules/BENZENE/BENZ_cog.itp",
    ],
    [...], # Other arguments
)
```

SIRAH force field

While COBY was primarily made for the Martini force field, it is still possible to create systems compatible with other CG force fields, such as SIRAH[11] (Fig. 2j). The SIRAH-compatible molecules used in system building (DMPC, water, Na and Cl) were imported from the pdb files using the `molecule_import` argument.

Ionic liquid

Finally, although aqueous solvents are by far most frequent due to their ubiquity in biological systems, COBY can be used to model other types solvents, such as ionic liquids. Various ionic liquids have been trialed in various (bio)industrial applications, such as catalysis,[17] cellulose degradation,[18] improved antibody delivery,[19] or as battery electrolytes. The created ionic liquid system (Fig. 2k) contained six different types of cations and one counterbalancing anion type. The algorithm is designed to insert molecules in order of size (starting from the largest one), ensuring that all the required molecules were placed within the box without overlaps. The structures of cations and anions were imported using the `molecule_import` functionality.

References

(1) Ingólfsson, H. I.; Bhatia, H.; Zeppelin, T.; Bennett, W. F. D.; Carpenter, K. A.; Hsu, P.-C.; Dharuman, G.; Bremer, P.-T.; Schiøtt, B.; Lightstone, F. C.; Carpenter, T. S. Capturing Biologically Complex Tissue-Specific Membranes at Different Levels of Compositional Complexity. *J. Phys. Chem. B* **2020**, *124*, 7819–7829, doi: 10.1021/acs.jpcb.0c03368.

(2) Cuello, L. G.; Jogini, V.; Cortes, D. M.; Perozo, E. Structural mechanism of C-type inactivation in K+ channels. *Nature* **2010**, *466*, 203–208, doi: 10.1038/nature09153.

(3) Kutzner, C.; Köpfer, D. A.; Machtens, J.-P.; de Groot, B. L.; Song, C.; Zachariae, U. Insights into the function of ion channels by computational electrophysiology simulations. *Biochim Biophys. Acta.* **2016**, *1858*, 1741–1752, doi: 10.1016/j.bbamem.2016.02.006.

(4) Hammond, A. T.; Heberle, F. A.; Baumgart, T.; Holowka, D.; Baird, B.; Feigenson, G. W. Crosslinking a lipid raft component triggers liquid ordered-liquid disordered phase separation in model plasma membranes. *Proc. Natl. Acad. Sci. U. S. A.* **2005**, *102*, 6320–6325, doi: 10.1073/pnas.0405654102.

(5) Saud, Z.; Tyrrell, V. J.; Zaragkoulias, A.; Protty, M. B.; Statkute, E.; Rubina, A.; Bentley, K.; White, D. A.; Rodrigues, P. D. S.; Murphy, R. C.; Köfeler, H.; Griffiths, W. J.; Alvarez-Jarreta, J.; Brown, R. W.; Newcombe, R. G.; Heyman, J.; Pritchard, M.; Mcleod, R. W.; Arya, A.; Lynch, C.-A.; Owens, D.; Jenkins, P. V.; Buurma, N. J.; O'Donnell, V. B.; Thomas, D. W.; Stanton, R. J. The SARS-CoV2 envelope differs from host cells, exposes procoagulant lipids, and is disrupted in vivo by oral rinses. *J. Lipid Res.* **2022**, *63*, 100208, doi: 10.1016/j.jlr.2022.100208.

(6) Su, R.; Zeng, J.; Marcink, T. C.; Porotto, M.; Moscona, A.; O'Shaughnessy, B. Host Cell Membrane Capture by the SARS-CoV-2 Spike Protein Fusion Intermediate. *ACS Cent. Sci.* **2023**, *9*, 1213–1228, doi: 10.1021/acscentsci.3c00158.

(7) Pedebos, C.; Smith, I. P. S.; Boags, A.; Khalid, S. The hitchhiker's guide to the periplasm: Unexpected molecular interactions of polymyxin B1 in E. coli. *Structure* **2021**, *29*, 444–456.e2, doi: 10.1016/j.str.2021.01.009.

(8) Uusitalo, J. J.; Ingólfsson, H. I.; Akhshi, P.; Tieleman, D. P.; Marrink, S. J. Martini Coarse-Grained Force Field: Extension to DNA. *Journal of Chemical Theory and Computation* **2015**, *11*, 3932–3945, doi: 10.1021/acs.jctc.5b00286.

(9) Corsi, J.; Hawtin, R. W.; Ces, O.; Attard, G. S.; Khalid, S. DNA Lipoplexes: Formation of the Inverse Hexagonal Phase Observed by Coarse-Grained Molecular Dynamics Simulation. *Langmuir* **2010**, *26*, 12119–12125, doi: 10.1021/la101448m.

(10) Marcink, T. C.; Simoncic, J. A.; An, B.; Knapinska, A. M.; Fulcher, Y. G.; Akkaladevi, N.; Fields, G. B.; Van Doren, S. R. MT1-MMP Binds Membranes by Opposite Tips of Its β Propeller to Position It for Pericellular Proteolysis. *Structure* **2019**, *27*, 281–292.e6, doi: 10.1016/j.str.2018.10.008.

(11) Klein, F.; Soñora, M.; Santos, L. H.; Frigini, E. N.; Ballesteros-Casallas, A.; Machado, M. R.; Pantano, S. The SIRAH force field: A suite for simulations of complex biological systems at the coarse-grained and multiscale levels. *J. Struct. Biol.* **2023**, *215*, 107985, doi: 10.1016/j.jsb.2023.107985.

(12) Hunter, J. C.; Gurbani, D.; Ficarro, S. B.; Carrasco, M. A.; Lim, S. M.; Choi, H. G.; Xie, T.; Marto, J. A.; Chen, Z.; Gray, N. S.; Westover, K. D. In situ selectivity profiling and crystal structure of SML-8-73-1, an active site inhibitor of oncogenic K-Ras G12C. *Proc. Natl. Acad. Sci. U. S. A.* **2014**, *111*, 8895–8900, doi: 10.1073/pnas.1404639111.

(13) Bussi, G.; Donadio, D.; Parrinello, M. Canonical sampling through velocity rescaling. *Journal of Chemical Physics* **2007**, *126*, doi: 10.1063/1.2408420.

(14) Bernetti, M.; Bussi, G. Pressure control using stochastic cell rescaling. *The Journal of Chemical Physics* **2020**, *153*, doi: 10.1063/5.0020514.

(15) Bereau, T.; Kremer, K. Automated Parametrization of the Coarse-Grained Martini Force Field for Small Organic Molecules. *Journal of Chemical Theory and Computation* **2015**, *11*, 2783–2791, doi: 10.1021/acs.jctc.5b00056.

(16) Tan, Y. S.; Spring, D. R.; Abell, C.; Verma, C. S. The Application of Ligand-Mapping Molecular Dynamics Simulations to the Rational Design of Peptidic Modulators of Protein-Protein Interactions. *Journal of Chemical Theory and Computation* **2015**, *11*, 3199–210, doi: 10.1021/ct5010577.

(17) McNeice, P.; Marr, P. C.; Marr, A. C. Basic ionic liquids for catalysis: The road to greater stability. *Catalysis Science and Technology* **2021**, *11*, 726–741, doi: 10.1039/d0cy02274h.

(18) Swatloski, R. P.; Spear, S. K.; Holbrey, J. D.; Rogers, R. D. Dissolution of Cellose with Ionic Liquids. *Journal of the American Chemical Society* **2002**, *124*, 4974–5, doi: 10.1021/ja025790m.

(19) Angsantikul, P.; Peng, K.; Curreri, A. M.; Chua, Y.; Chen, K. Z.; Ehondor, J.; Mitragotri, S. Ionic Liquids and Deep Eutectic Solvents for Enhanced Delivery of Antibodies in the Gastrointestinal Tract. *Advanced Functional Materials* **2021**, *31*, 2002912, doi: 10.1002/adfm.202002912.
